# Influent factors of gestational vitamin D deficiency and its relation to an increased risk of preterm delivery in Chinese population

**DOI:** 10.1038/s41598-018-21944-3

**Published:** 2018-02-26

**Authors:** Yuan-Hua Chen, Lin Fu, Jia-Hu Hao, Hua Wang, Cheng Zhang, Fang-Biao Tao, De-Xiang Xu

**Affiliations:** 1 School of Public Health, Anhui Medical University, Hefei, 230032 China; 2 School of Basic Medical Science, Anhui Medical University, Hefei, 230032 China; 3 Anhui Provincial Key Laboratory of Population Health & Aristogenics, Anhui Medical University, Hefei, 230032 China

## Abstract

Gestational vitamin D deficiency (VDD) has been linked with adverse pregnant outcomes. To investigate influent factors of gestational VDD and its relation to the incidence of preterm delivery, total 3598 eligible mother-and-singleton-offspring pairs were recruited. For serum 25(OH)D concentration, 941 pregnant women were sufficient, 1260 insufficient, and 1397 deficient. Further analysis showed that VDD was more prevalent in winter than in other seasons. Underweight but not overweight was a risk factor for gestational VDD. Multivitamin use reduced risk of gestational VDD. Interestingly, 8.23% delivered preterm infants among subjects with VDD (adjusted RR: 4.02; 95% CI: 2.33, 6.92) and 3.81% among subjects with gestational vitamin D insufficiency (VDI) (adjusted RR: 2.07; 95% CI: 1.16, 3.71). Moreover, 2.59% delivered early preterm infants among subjects with VDD (adjusted RR: 2.97; 95% CI: 1.41, 6.24) and 0.49% among subjects with VDI (adjusted RR: 0.54; 95% CI: 0.19, 1.51). The incidence of late preterm delivery was 5.64% among subjects with VDD (adjusted RR: 3.90; 95% CI: 2.26, 6.72) and 3.32% among subjects with VDI (adjusted RR: 2.09; 95% CI: 1.17, 3.74). In conclusion, pre-pregnancy BMI, seasonality and multivitamin use are influent factors of gestational vitamin D status. Gestational VDD is associated with an increased risk of preterm delivery in Chinese population.

## Introduction

Vitamin D, a secosteroid hormone, is synthesized primarily in the skin upon exposure to sunlight and is converted to active vitamin D3 in the liver and the kidney. The classical function of vitamin D is regulating calcium and phosphorus homeostasis^1^. Recently, vitamin D is well recognized for its non-classical actions including antioxidant activity, modulation of innate immune response and adaptive immune response^2–4^. Vitamin D deficiency (VDD), defined as serum 25(OH)D level <20 ng/ml, is very prevalent in worldwide and affects people of all age, especially women of childbearing age^5^. Although some potential influent factors for vitamin D status, such as seasonality, age, smoking, race and ethnicity have been identified in several case-control studies^6–8^, research reports on the influent factors for gestational vitamin D status in a population-based cohort are lacking.

Several epidemiological investigations have demonstrated that gestational VDD elevates risk of pregnancy complications including preeclampsia^9,10^. In contrast, the reports from randomized controlled trials indicate that gestational vitamin D supplementation can markedly decrease pregnancy complications including preeclampsia and pregnancy induced hypertension^11^. Moreover, gestational VDD has been linked with the impaired neurobehavioral development, the increased asthma and schizophrenia in adult offspring^12,13^. A recent study suggests that gestational VDD elevates risks of small for gestational age and low birth weight infants^14^. On the other hand, animal experiments demonstrate that gestational VDD results in reproductive dysfunction and impairment of neurobehavioral development in adult offspring^15,16^. By contrast, vitamin D3 supplementation protects mice from lipopolysaccharide-induced fetal intrauterine growth restriction and neural tube defects^17,18^.

Preterm delivery, defined as spontaneous or iatrogenic delivery before gestational week 37, is a major reason for neonatal deaths^19^. Several studies explored the association between gestational VDD and preterm delivery with contradictory results^20–23^. A case-cohort study from the US Collaborative Perinatal Project showed that gestational VDD elevated risk of preterm delivery among nonwhite women but not white women, indicating a racial disparity on the link between VDD and preterm delivery^24^. The objective of the present study was to analyze influent factors of gestational VDD and its relation to the incidence of preterm delivery in Chinese population.

## Results

### Demographic characteristics of pregnant women

No subjects were suffering from preeclampsia, gestational diabetes, maternal drug uses, smoking cigarette and drinking alcohol throughout pregnancy (data not shown) in this study. Serum 25(OH)D concentration was measured among 3598 pregnant women. The mean maternal serum 25(OH)D concentration was 24.91 ± 9.30 (±SD) ng/ml in this cohort. For 25(OH)D concentration, only 941 pregnant women (26.15%) were sufficient, 1260 (35.02%) insufficient, and 1397 (38.83%) deficient (Table 1). The demographic characteristics of pregnant women and their newborns were compared among subjects with sufficiency, insufficiency and deficiency. No significant difference on maternal age, family monthly income, gestational week of blood sample and parity was observed among three groups (Table 1). There was a significant difference on pre-pregnancy BMI, season of blood sample and periconceptional multivitamin use among three groups (Table 1).

**Table 1:** Demographic characteristics of 3598 mothers.

Demographic variables	Gestational vitamin D status^1^			*P*-value^2^
	Deficiency	Insufficiency	Sufficiency	
Pregnant women [n (%)]	1397 (38.83)	1260 (35.02)	941 (26.15)	
Maternal age [years, n (%)]				
<25 25–34 ≥35	220 (15.75) 1131 (80.96) 46 (3.29)	194 (15.40) 1022 (81.11) 44 (3.49)	147 (15.62) 768 (81.61) 26 (2.77)	0.909
Maternal BMI [kg/m^2^, n (%)]				
Underweight (<18.5) Normal weight (18.5–22.9) Overweight (≥23.0)	333 (23.84) 925 (66.21) 139 (9.95)	271 (21.51) 859 (68.17) 130 (10.32)	168 (17.85) 649 (68.97) 124 (13.18)	0.003
Season of blood sample [n (%)]				
Spring Summer Fall Winter	477 (34.14) 311 (22.26) 279 (19.97) 330 (23.62)	468 (37.14) 271 (21.51) 275 (21.83) 246 (19.52)	371 (39.43) 225 (23.91) 193 (20.51) 152 (16.15)	0.001
Periconceptional multivitamin use [n (%)]				
No Less than one month More than one month	1220 (87.33) 97 (6.94) 80 (5.73)	1033 (81.98) 112 (8.89) 115 (9.13)	743 (78.96) 81 (8.61) 117 (12.43)	<0.001
Family monthly income (RMB/yuan) [n (%)]				
Low (<2000) Middle (2000–3999) High (≥4000)	611 (43.74) 590 (42.23) 196 (14.03)	557 (44.21) 516 (40.95) 187 (14.84)	432 (45.91) 372 (39.53) 137 (14.56)	0.746
Parity [n(%)]				
1 >1	1345 (96.87) 60 (3.13)	1238 (96.97) 51 (3.03)	927 (96.58) 37 (3.42)	0.854
Gestational week of blood sample [w, n (%)]				
First-trimester (<13) Second-trimester (13–27)	519 (37.15) 878 (62.85)	444 (35.24) 816 (64.76)	337 (35.81) 604 (64.19)	0.575

^1^25(OH)D < 20 ng/ml for deficiency; 20 ≤ 25(OH)D < 30 ng/ml for insufficiency; 25(OH)D ≥ 30 ng/ml for sufficiency.

^2^Differences among groups were assessed with a chi-square test for categorical variables.

### Influence of demographic characteristics on gestational serum 25(OH)D concentration

**Figure 1:**
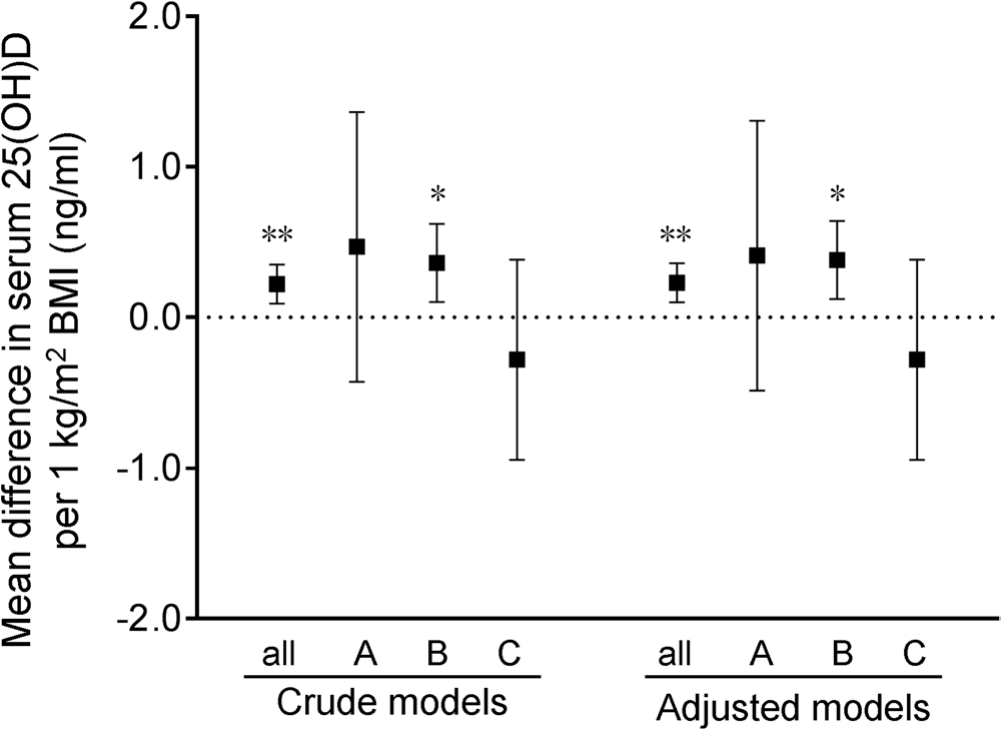
Association between maternal pre-pregnancy BMI and serum 25(OH)D concentration based on linear regression analyses. (**A**–**C**) Stratification analyses by pre-pregnancy BMI. (**A**) Underweight (<18.5 kg/m^2^); (**B**) normal weight (18.5–22.9 kg/m^2^); (**C**) overweight (≥23.0 kg/m^2^). ^*^P < 0.05, ^**^P < 0.01.

The demographic characteristics for influencing gestational vitamin D status were analyzed. As shown in Table 2, maternal age, family monthly income, parity and gestational week of blood sample, did not influence gestational serum 25(OH)D concentration. As expected, gestational serum 25(OH)D concentration was higher in spring and summer than in winter (Table 2). Serum 25(OH)D concentration was increased among multivitamin users (Table 2). In addition, serum 25(OH)D concentration was slightly higher among subjects with either normal weight or overweight than those of subjects with underweight (Table 2). The association between maternal pre-pregnancy BMI and serum 25(OH)D concentration were then analyzed based on linear regression analyses. As shown in Fig. 1, for crude models, mean differences in serum 25(OH)D concentration per 1 kg/m^2^ pre-pregnancy BMI were 0.22 ng/ml (95% CI: 0.09, 0.35) among all subjects, 0.47 ng/ml (95% CI: −0.42, 1.37) among subjects with underweight, 0.36 ng/ml (95% CI: 0.10, 0.62) among subjects with normal weight, −0.28 ng/ml (95% CI: −0.94, 0.39) among subjects with overweight, respectively. After adjustment for maternal age, periconceptional multivitamin use, and seasonality, mean differences in 25(OH)D per 1 kg/m^2^ pre-pregnancy BMI were 0.23 ng/ml (95% CI: 0.10, 0.36) among all subjects, 0.41 ng/ml (95% CI: −0.49, 1.30) among subjects with underweight, 0.38 ng/ml (95% CI: 0.12, 0.64) among subjects with normal weight, −0.28 ng/ml (95% CI: −0.94, 0.39) among subjects with overweight, respectively.

**Table 2:** Influence of demographic characteristics on serum 25(OH)D level.

Characteristics	n (%)	Serum 25(OH)D (ng/ml, means ± SD)	*P*-value^2^
Age [years]			
<25 25–34 ≥ 35	561 (15.59) 2921 (81.19) 116 (3.22)	24.92 ± 9.43 24.91 ± 9.29 24.6 ± 9.24	0.971
Pre-pregnancy BMI [kg/m^2^]			
Underweight (<18.5) Normal weight (18.5–22.9) Overweight (≥23.0)	772 (21.46) 2433 (67.62) 393 (10.92)	24.20 ± 9.24 24.99 ± 9.30 25.80 ± 9.37	0.017
Season of blood sample [n (%)]^1^			
Spring Summer Fall Winter	1316 (36.58) 807 (22.43) 747 (20.76) 728 (20.23)	25.49 ± 9.67 25.36 ± 9.46 24.76 ± 8.69 23.50 ± 8.93	<0.001
Periconceptional multivitamin use [n (%)]			
No Less than one month More than one month	2996 (83.27) 290 (8.06) 312 (8.67)	24.57 ± 9.1 25.71 ± 9.28 27.00 ± 9.98	<0.001
Family monthly income (RMB/yuan) [n (%)]			
Low (<2000) Middle (2000–3999) High (≥4000)	1600 (44.47) 1478 (41.08) 520 (14.45)	25.17 ± 9.53 24.54 ± 9.03 25.14 ± 9.33	0.140
Parity [n(%)]			
1 >1	3450 (95.89) 148 (4.11)	24.93 ± 9.32 24.77 ± 9.56	0.833
Gestational week of blood sample [w, n (%)]			
First-trimester (<13) Second-trimester (13–27)	1300 (36.13) 2298 (63.87)	24.85 ± 9.46 24.94 ± 9.22	0.783

^1^Spring: March to May; Summer: June to August; Fall: September to November; Winter: December to February.

^2^ANOVA and the Student-Newmann-Keuls post hoc test were used to determine differences among different groups. Student *t* test was used to determine differences between two groups.

### Association between demographic characteristics and the risk of gestational VDD

The association between pre-pregnancy BMI and the risk of VDD was analyzed. As shown in Table 3, crude RR for VDD was 1.25 (95% CI: 1.06, 1.47) among underweight women, 0.84 (95% CI: 0.67, 1.05) among overweight women using multiple logistic regression model. After adjustment for maternal age, periconceptional multivitamin use, and seasonality, RR for VDD was 1.26 (95% CI: 1.07, 1.48) among underweight women and 0.84 (95% CI: 0.67, 1.06) among overweight women (Table 3). The association between seasonality and the risk of VDD was also analyzed. As shown in Table 3, crude RR for VDD was 1.11 (95% CI: 0.93, 1.33) among women during summer, 1.06 (95% CI: 0.88, 1.27) among women during fall, and 1.45 (95% CI: 1.21, 1.74) among women during winter using multiple logistic regression model. After adjustment for maternal age, periconceptional multivitamin use, and pre-pregnancy BMI, RR for VDD was 1.12 (95% CI: 0.94, 1.34) among women during summer, 1.05 (95% CI: 0.87, 1.27) among women during fall, and 1.45 (95% CI: 1.21, 1.74) among women during winter (Table 3). The association between periconceptional multivitamin use and the risk of VDD was analyzed. As shown in Table 3, crude RR for VDD was 0.69 (95% CI: 0.51, 0.94) among women with periconceptional multivitamin use less than one month, 0.72 (95% CI: 0.58, 0.89) among women with periconceptional multivitamin use more than one month using multiple logistic regression model. After adjustment for maternal age, pre-pregnancy BMI, and seasonality, RR for VDD was 0.68 (95% CI: 0.50, 0.93) among women with periconceptional multivitamin use less than one month and 0.72 (95% CI: 0.58, 0.90) among women with periconceptional multivitamin use more than one month (Table 3).

**Table 3:** Association between demographic characteristics and the risk of gestational vitamin D deficiency based on multiple logistic regression analyses.

Parameter	Crude models		Adjusted models	
	*RR* (95% *CI*)	*P* values	*RR* (95% CI)	*P* values
Pre-pregnancy BMI [kg/m^2^]^1^				
<18.5	1.25 (1.06, 1.47)	0.007	1.26 (1.07, 1.48)	0.006
18.5–22.9	1.00		1.00	
≥23.0	0.84 (0.67, 1.05)	0.116	0.84 (0.67, 1.06)	0.135
Season of blood sample^2^				
Spring	1.00		1.00	
Summer	1.11 (0.93, 1.33)	0.260	1.12 (0.94, 1.34)	0.211
Fall	1.06 (0.88, 1.27)	0.555	1.05 (0.87, 1.27)	0.601
Winter	1.45 (1.21, 1.74)	<0.001	1.45 (1.21, 1.74)	<0.001
Periconceptional multivitamin use^3^				
No	1.00		1.00	
Less than one month	0.69 (0.51, 0.94)	0.019	0.68 (0.50, 0.93)	0.016
More than one month	0.72 (0.58, 0.89)	0.002	0.72 (0.58, 0.90)	0.003

^1^Adjusted for maternal age, periconceptional multivitamin use, and season of sampling.

^2^Adjusted for maternal age, pre-pregnancy BMI, periconceptional multivitamin use.

^3^Adjusted for maternal age, pre-pregnancy BMI, and season of sampling.

### Association between gestational vitamin D status and the risk of preterm delivery

The association between gestational vitamin D status and the risk of preterm delivery was analyzed. As shown in Table 4, 8.23% delivered preterm infants among subjects with gestational VDD (RR: 3.28; 95% CI: 2.12, 5.11) and 3.81% among subjects with gestational VDI (RR: 1.45; 95% CI: 0.89, 2.37). After adjustment for maternal pre-pregnancy BMI, maternal age, periconceptional multivitamin use, and seasonality, RR for preterm delivery was 4.02 (95% CI: 2.33, 6.92) among subjects with VDD and 2.07 (95% CI: 1.16, 3.71) among subjects with VDI using multiple logistic regression model (Table 4).

**Table 4:** Crude and adjusted RRs for preterm delivery in different groups.

Parameter	Gestational vitamin D status		
	Sufficiency	Insufficiency	Deficiency
Gestational week (week)	39.3 ± 1.7	39.0 ± 1.5**	38.6 ± 2.5**^††^
Preterm delivery [n (%)]	25 (2.66)	48 (3.81)	115 (8.23)
Crude *RR* (95% *CI*)	1.00	1.45 (0.89, 2.37)	3.28 (2.12, 5.11)**
Adjusted *RR* (95% *CI*)^1^	1.00	2.07 (1.16, 3.71)*	4.02 (2.33, 6.92)**^††^
Early preterm delivery [n (%)]^2^	9 (0.97)	6 (0.49)	34 (2.59)
Crude *RR* (95% *CI*)	1.00	0.50 (0.18, 1.42)	2.70 (1.29, 5.66)**^††^
Adjusted *RR* (95% *CI*)^1^	1.00	0.54 (0.19, 1.51)	2.97 (1.41, 6.24)**^††^
Late preterm delivery [n (%)]2	16 (1.69)	42 (3.32)	81 (5.64)
Crude *RR* (95% *CI*)	1.00	1.98 (1.11, 3.55)*	3.62 (2.10, 6.23)**^††^
Adjusted *RR* (95% *CI*)^1^	1.00	2.09 (1.17, 3.74)*	3.90 (2.26, 6.72)**^††^

^1^Adjusted for pre-pregnancy BMI, maternal age, periconceptional multivitamin use, and season of sampling.

^2^Gestational week <32 weeks for early preterm delivery; 32≤ gestational week <37 weeks for late preterm delivery.

^*^P < 0.05, **P < 0.01 as compared with sufficiency; ^††^*P* < 0.01 as compared with insufficiency.

The association between gestational vitamin D status and risk of early preterm delivery and late preterm delivery were then analyzed. As shown in Table 4, the incidence of early preterm delivery was 2.59% among subjects with gestational VDD (RR: 2.70; 95% CI: 1.29, 5.66) and 0.49% among subjects with gestational VDI (RR: 0.50; 95% CI: 0.18, 1.42). After adjustment for maternal pre-pregnancy BMI, maternal age, periconceptional multivitamin use, and seasonality, RR for early preterm delivery was 2.97 (95% CI: 1.41, 6.24) among subjects with VDD and 0.54 (95% CI: 0.19, 1.51) among subjects with VDI using multiple logistic regression model (Table 4). The incidence of late preterm delivery was 5.64% among subjects with VDD (RR: 3.62; 95% CI: 2.10, 6.23) and 3.32% among subjects with VDI (RR: 1.98; 95% CI: 1.11, 3.55). After adjustment for maternal pre-pregnancy BMI, maternal age, periconceptional multivitamin use, and seasonality, RR for late preterm delivery was 3.90 (95% CI: 2.26, 6.72) among subjects with VDD and 2.09 (95% CI: 1.17, 3.74) among subjects with VDI using multiple logistic regression model (Table 4).

## Discussion

The present study analyzed vitamin D status among 3598 pregnant women. For serum 25(OH)D concentration, only 26.15% pregnant women were sufficient, 35.02% insufficient, and 38.83% deficient. These results are in agreement with the results from several recent studies^5,25^. In this cohort, no subject was suffering from preeclampsia, smoking cigarette and drinking alcohol during pregnancy (data not shown). A report demonstrated that socio-economic status influences gestational vitamin D status^26^. In addition, age, parity, smoking and seasonality are important determinants of gestational serum 25(OH)D concentration^27^. Thus, the present study analyzed the effects of maternal age, family monthly income, parity, gestational week of blood sample on gestational serum 25(OH)D concentration. Results show that these factors did not affect gestational vitamin D status. The present study also investigated the association pre-pregnancy BMI with serum 25(OH)D concentration. Our results demonstrated that the mean 25(OH)D concentration was lower among subjects with underweight than those of subjects with either normal weight or overweight. Pre-pregnancy BMI, as a predictor of serum 25(OH)D, each additional 1 kg/m^2^ BMI was associated with an additional 0.23 ng/ml serum 25(OH)D based on linear regression analyses. Moreover, the association between pre-pregnancy BMI and the risk of VDD was then analyzed. Adjusted RR for VDD was 1.26 (95% CI: 1.07, 1.48) among underweight women and 0.84 (95% CI: 0.67, 1.06) among overweight women using multiple logistic regression model. Taken together, these results suggest that underweight but not overweight is a risk factor for gestational VDD. However, some earlier reports from non-Asian countries demonstrated that obesity was a risk factor for VDD^28^. The cause of the inconsistency may be due to the different dietary structure between non-Asian countries and Asian countries. Several studies showed that multivitamin use can improve their vitamin D status^29,30^. In the present study, the mean serum 25(OH)D concentration was markedly increased among multivitamin users. Adjusted RR for VDD was 0.68 (95% CI: 0.50, 0.93) among women with periconceptional multivitamin use less than one month and 0.72 (95% CI: 0.58, 0.90) among women with periconceptional multivitamin use more than one month, respectively. These results indicated that periconceptional multivitamin use is associated with a decreased risk of gestational VDD. Numerous studies showed that season is a major influent factor for vitamin D status^31,32^. In the present study, gestational serum 25(OH)D concentration was higher in spring and summer than in winter. In addition, gestational VDD was more prevalent in winter than in other seasons.

It remains contradictory whether VDD elevates the risks of preterm infants. According to a nested case-control study and a prospective cohort study, gestational VDD was not associated with spontaneous preterm delivery^20,21^. By contrast, the data from prospective cohort studies and observational studies indicated that gestational VDD elevates the risk of preterm infants^22,23,33^. An early report showed that optimal conversion of circulating 25(OH)D to 1,25(OH)2D3, the active hormone, occurs around 40 ng/ml^34^. Follow-up, several randomized trial of vitamin D supplementation found that the rate of preterm delivery is significant more lower in pregnant women with serum 25(OH)D ≥40 ng/ml compared to those with serum 25(OH)D ≤20 ng/ml, suggesting higher 25(OH)D concentration was associated with an decreased risk of preterm delivery^35–38^. The inconsistency of past findings may be related to following reasons: first, cohort among pregnant women with relatively vitamin D replete, with a history of preterm delivery or at high risk for preeclampsia; second, gestational vitamin D status is a link to ethnic disparities in adverse birth outcomes^24^. Although a recent study in northeast China demonstrated that VDD was more prevalent in the severe preterm group than in the mildly preterm group and the in-term group, they did not analyze the association between VDD and an increased risk of preterm delivery because vitamin D status at prior to labor cannot be used to assess risk of preterm delivery^39^. The present study analyzed the association between vitamin D status during first- and second-trimester and preterm delivery in Chinese population. The RR for preterm delivery was 3.52 among subjects with VDD and 1.53 among subjects with VDI as compared with those with VDS. Adjusted RR for preterm infants was 3.87 among subjects with VDD and 1.58 among subjects with VDI, suggesting that VDD elevates the risks of preterm infants in Chinese population.

Until now, few reports analyzed the association between VDD and risk of early or late preterm delivery. The present study found that the incidence of early preterm delivery was 2.59% among subjects with VDD, 0.49% among subjects with VDI and 0.97% among subjects with VDS. Adjusted RR for early preterm delivery was 2.97 among subjects with VDD and 0.54 among subjects with VDI, indicating that VDD but not VDI elevates the risks of early preterm infants. In addition, the incidence of late preterm delivery was 5.64% among subjects with VDD, 3.32% among subjects with VDI and 1.69% among subjects with VDS. Adjusted RR for late preterm delivery was 3.90 among subjects with VDD and 2.09 among subjects with VDI, indicating that not only VDD but also VDI elevates the risks of late preterm infants.

The present study laid emphasis on influent factor of VDD and whether VDD elevates risk of preterm delivery in Chinese population. The present study has several limitations. First, only a single sample at different gestational ages was analyzed in the present study. Second, the present study did not clarify the mechanism why VDD elevated the risks of preterm delivery. Increasing evidence demonstrates that vitamin D has an anti-inflammatory activity^40^. Recently, an animal report indicates that vitamin D inhibits placental inflammation through reinforcing physical interaction between vitamin D receptor (VDR) and NF-κB p65 subunit^17^. Several case-control studies also found that the percentage of VDR-positive nucleus in the prostate was decreased in cases. By contrast, the percentage of NF-κB p65-positive nucleus was increased in cases^41^. Additional analysis showed that serum 25(OH)D level was negatively associated with serum inflammatory molecules levels, such as Interleukin (IL)-8 and C-reactive protein (CRP), in cases^41,42^. Indeed, placental inflammation has been associated with adverse pregnant outcomes including preterm delivery^43,44^. The exact mechanism by which VDD induces preterm delivery needs to be explored in animal experiments.

In summary, the present study analyzed influent factors of gestational VDD. The present results demonstrate that VDD is more prevalent in winter than in other seasons. Underweight but not overweight is a risk factor for VDD. Multivitamin use is associated with a decreased risk of VDD. The present study suggests that VDD elevates the risks of preterm infants in Chinese population. Moreover, VDD but not VDI elevates the risks of early preterm infants. Not only VDD but also VDI elevates the risks of late preterm infants.

## Methods

### Participants

**Figure 2:**
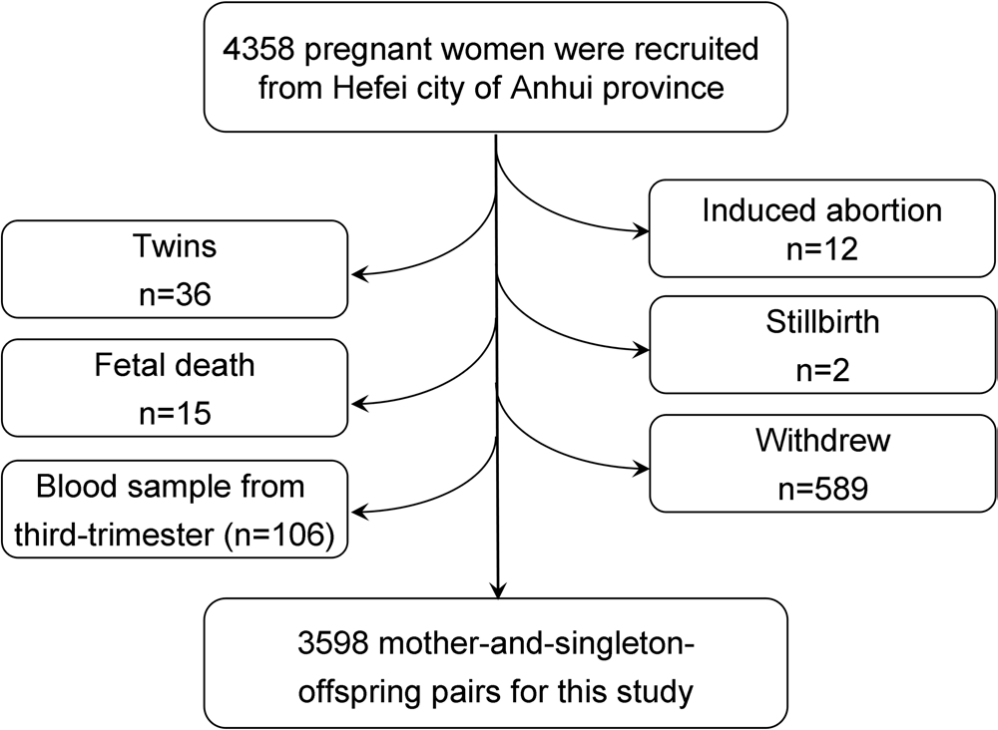
Flow diagram of recruitment and follow-up in this birth cohort study.

The present study is a prospective population-based cohort study that recruited 4358 pregnant women from Hefei city from January 1 to December 31 in 2009. Exclusion criteria were as follows: inability to answer questions in Chinese, inability to provide informed consent, mental disorders, and pregnancy complications (pregnancy induced hypertension, preeclampsia, and gestational diabetes), or plans to leave local places before delivery. For this study, total 3598 mother-and-singleton-offspring pairs were eligible (Fig. 2). The present study obtained ethics approval from the ethics committee of Anhui Medical University (No. 2008020). All participants signed a written informed consent for this study. The methods were carried out in accordance with the approved guidelines.

### Definition of preterm delivery

Preterm delivery was defined as delivery before 37 gestational weeks, or fewer than 259 days since the first day of the women’s last menstrual period (Gestational week <32 weeks for early preterm delivery and 32≤ gestational week <37 weeks for late preterm delivery)^19,45^.

### Measurement of 25(OH)D

Maternal non-fasting blood samples taken as part of routine antenatal care were collected and stored at −80 °C, with no further freeze-thaw cycles, until 25(OH)D measurement. Serum 25(OH)D was measured by Radioimmunoassay (RIA) using a kit from Diasorin (DiaSorin Inc, Stillwater, MN, USA) following manufacturer’s instructions^14,46^. Gestational vitamin D status was divided into three groups according to following criteria: 25(OH)D <20 ng/ml for VDD, 20 ≤ 25(OH)D <30 ng/ml for vitamin insufficiency (VDI), and 25(OH)D ≥30 ng/ml for vitamin D sufficiency (VDS)^5,14^.

### Statistical analysis

Differences between included mother-and-offspring pairs and those excluded because of missing data were investigated with *t* tests for continuously measured variables (with those variables that were right-skewed being logged) and χ^2^ tests for categorical variables. Multiple logistic regression model was used to estimate RR with 95% confidence intervals (95% CI). ANOVA and the Student-Newmann-Keuls post hoc test were used to determine differences among different groups. Student *t* test was used to determine differences between two groups.
